# Analysis of pre- and intraoperative clinical for successful operating room extubation after living donor liver transplantation: a retrospective observational cohort study

**DOI:** 10.1186/s12871-019-0781-z

**Published:** 2019-06-28

**Authors:** Min Suk Chae, Jong-Woan Kim, Joon-Yong Jung, Ho Joong Choi, Hyun Sik Chung, Chul Soo Park, Jong Ho Choi, Sang Hyun Hong

**Affiliations:** 1 Department of Anesthesiology and Pain medicine, Seoul St. Mary’s Hospital, College of Medicine, The Catholic University of Korea, 222, Banpo-daero, Seocho-gu, Seoul, 06591 Republic of Korea; 2 Department of Radiology, Seoul St. Mary’s Hospital, College of Medicine, The Catholic University of Korea, Seoul, Republic of Korea; 3 Department of Surgery, Seoul St. Mary’s Hospital, College of Medicine, The Catholic University of Korea, Seoul, Republic of Korea

**Keywords:** Psoas muscles, Liver transplantation, Airway extubation, Operating rooms

## Abstract

**Background:**

Early extubation after liver transplantation is safe and accelerates patient recovery. Patients with end-stage liver disease undergo sarcopenic changes, and sarcopenia is associated with postoperative morbidity and mortality. We investigated the impact of core muscle mass on the feasibility of immediate extubation in the operating room (OR) after living donor liver transplantation (LDLT).

**Methods:**

A total of 295 male adult LDLT patients were retrospectively reviewed between January 2011 and December 2017. In total, 40 patients were excluded due to emergency surgery or severe encephalopathy. A total of 255 male LDLT patients were analyzed in this study. According to the OR extubation criteria, the study population was classified into immediate and conventional extubation groups (39.6 vs. 60.4%). Psoas muscle area was estimated using abdominal computed tomography and normalized by height squared (psoas muscle index [PMI]).

**Results:**

There were no significant differences in OR extubation rates among the five attending transplant anesthesiologists. The preoperative PMI correlated with respiratory performance. The preoperative PMI was higher in the immediate extubation group than in the conventional extubation group. Potentially significant perioperative factors in the univariate analysis were entered into a multivariate analysis, in which preoperative PMI and intraoperative factors (i.e., continuous renal replacement therapy, significant post-reperfusion syndrome, and fresh frozen plasma transfusion) were associated with OR extubation. The duration of ventilator support and length of intensive care unit stay were shorter in the immediate extubation group than in the conventional extubation group, and the incidence of pneumonia and early allograft dysfunction were also lower in the immediate extubation group.

**Conclusions:**

Our study could improve the accuracy of predictions concerning immediate post-transplant extubation in the OR by introducing preoperative PMI into predictive models for patients who underwent elective LDLT.

**Electronic supplementary material:**

The online version of this article (10.1186/s12871-019-0781-z) contains supplementary material, which is available to authorized users.

## Background

Patients with end-stage liver disease (ESLD) frequently suffer from sarcopenia, where sarcopenia before liver transplantation (LT) is one of major risk factors for postoperative morbidity and mortality [1, 2]. A recent study revealed that the degree of perioperative core muscular loss is significantly associated with poor overall patient survival in living donor liver transplantation (LDLT) [3]. Because the model for end-stage liver disease (MELD) score has a limitation in terms of reflecting the physical and nutritional conditions of patients with ESLD, sarcopenia has additional prognostic value for morbidity and mortality in patients with ESLD [4–7]. Particularly, a MELD-Sarcopenia model proved superior to the MELD score in terms of predicting waiting list mortality in LT waiting-list patients with a low MELD score [8].

After LT, prolonged ventilator care with sedation in the intensive care unit (ICU) has been the typical postoperative management strategy [9]. However, studies of other surgeries showed that early tracheal extubation has favorable effects on postoperative patient recovery [10, 11]. Additionally, because the use of ventilator support in the ICU accounts for a large proportion of LT costs, successful early extubation may effectively reduce the financial burden [12]. However, although there is much evidence that early extubation after LT is a safe and feasible practice [12–18], many transplant centers still use routine mechanical ventilation in the ICU after LT. Because LT surgery is one of the most complex procedures currently performed, some transplant clinicians remain concerned regarding the potential risk of cardiopulmonary complications, reoperation, failed extubation, and impaired recovery from surgical stress [13, 19], despite the identification in previous studies of predictors of early extubation in the operating room (OR) after LT [14, 15].

The aim of this study was to investigate the association between pre- and intraoperative factors, including core muscle mass, and immediate extubation in the OR after LDLT. In addition, we compared short-term postoperative complications and outcomes according to OR extubation.

## Patients and methods

### Ethical considerations

The Institutional Review Board of Seoul St. Mary’s Hospital Ethics Committee approved this study for LDLT recipients (KC18RESI0205) on April 13, 2018, and it was performed according to the principles of the Declaration of Helsinki. The requirement for informed consent was waived due to the retrospective study design.

### Inclusion and exclusion criteria

The inclusion criteria were 1) male; 2) adult (age ≥ 19 years); and 3) patients who underwent elective LDLT. The clinical exclusion criteria were 1) emergency LDLT and 2) severe encephalopathy (West-Haven criteria III or IV) [20], because patients with those conditions underwent routine mechanical ventilation after surgery to protect the airway from pulmonary aspiration. Recipients or donors whose electronic medical records contained defective or missing data were also excluded.

### Living donor liver transplantation

Surgery and anesthesia were consistently provided by expert transplant surgeons and anesthesiologists with > 5 years’ experience in LDLT, respectively. The surgical procedure and anesthetic management were described in detail in our previous studies [3, 21, 22]. Briefly, the piggyback technique was performed using the right liver lobe with reconstruction of the middle hepatic vein. Following the hepatic vessel and bile duct anastomoses, patency of the hepatic vessels was confirmed by Doppler ultrasonography.

Balanced anesthesia was performed under multiple invasive monitoring. The optimal hemodynamic adjustment was made with a mean arterial pressure (MAP) of ≥65 mmHg and a central venous pressure of ≤10 mmHg. According to the Practice Guidelines for Perioperative Blood Management [23], packed red blood cells (PRBCs) were transfused to a hematocrit level of ≥25%, and coagulation factors were replaced as determined by laboratory assessment or thromboelastography (Thromboelastograph Model 5000; Haemoscope Corporation, Niles, IL, USA) [24]. The kidney function of patients who scheduled elective LDLT was regularly monitored by nephrologists, and patients with severely decreased kidney function before surgery (an increase in serum creatinine to ≥4.0 mg.dL^− 1^ or to 3-fold baseline level, a urine output of ≤0.3 mL.kg^− 1^.h^− 1^ for 24 h, or anuria for 12 h) were intraoperatively given continuous renal replacement therapy (CRRT) (PRISMAFLEX System; Baxter) [25–27]. The expert transplant anesthesiologists classified post-reperfusion syndrome (PRS) as significant, immediately after graft reperfusion, when unstable and persistent vital signs (i.e., hypotension ≥30% in the anhepatic phase or hypotensive duration ≥5 min); fatal arrhythmias (i.e., asystole or ventricular tachycardia); requirement for strong rescue vasopressors (i.e., epinephrine or norepinephrine infusion); continuing or reoccurring fibrinolysis; or a requirement for an anti-fibrinolytic drug were present [28].

An immunosuppression regimen (calcineurin inhibitor, mycophenolate mofetil, and prednisolone) was applied according to the hospital LDLT protocol. Basiliximab was administered before transplant surgery and 4 days after the surgery. The immunosuppressive drugs were progressively discontinued after LDLT.

### Criteria for immediate extubation in the operating room

Immediate extubation was defined as tracheal extubation in the OR at the end of surgery, and conventional extubation was defined as tracheal extubation in the ICU. The five attending anesthesiologists (M.S.C.; H.S.C.; C.S.P.; J.H.C.; S.H.H.), who specialized in anesthetic management for LT, decided whether to extubate patients in the OR, immediately after surgery, based on standardized and universally accepted criteria: adequate oxygenation (SpO_2_ ≥ 95%, with FiO_2_ ≤ 0.5); adequate ventilation (tidal volume ≥ 5 mL.kg^− 1^ and spontaneous respiration rate < 25 min^− 1^ with normocarbia [ETCO_2_ 30–40 mmHg]); stable hemodynamic condition or minimal use of a vasopressor (norepinephrine infusion < 0.1 μg.kg^− 1^.min^− 1^); full clinical reversal of muscle relaxation (sustained head lift for 5 s or hand grasp); neurologically intact condition (able to follow simple verbal orders, spontaneous eye opening, and proper cough/gag reflex); appropriate metabolic status (pH > 7.25, normal electrolytes, and euvolemia); normothermia (≥ 35.5 °C); and no surgical concerns regarding ongoing bleeding or hepatic vascular patency [12, 15, 17]. The surgeon was not consulted unless a surgical issue arose. All patients were transferred to the ICU after surgery.

The patients were classified into two groups: those who were extubated in the OR were classified into the immediate extubation group and those who were not extubated in the OR were classified into the conventional extubation group.

### Measurement of psoas muscle area

The abdominal condition of patients who were scheduled for elective LDLT was routinely investigated using computed tomography (CT) within 1 month prior to surgery.

The cross-sectional areas of both PMAs between lumbar vertebrae 3 and 4 were manually evaluated on abdominal CT images (PACS Viewer; INFINITT Healthcare Co., Ltd., Phillipsburg, NJ, USA) using a two-dimensional module, with intramuscular fatty infiltration removed from the PMA images using automated software (AQI; TeraRecon, Foster City, CA, USA). The average of the two PMAs was estimated and normalized to the patient’s height squared (PMI = PMA.height^− 2^). The abdominal CT images were analyzed by a radiologist (J.Y.J.) with 10 years’ experiences who was blinded to the clinical data.

In this study, the PMI was considered a core muscle index in patients who underwent elective LDLT [3, 29, 30].

### Correlations between respiratory performance and preoperative PMI

We studied the correlations between respiratory performance using preoperative spirometry parameters (i.e., forced vital capacity [FVC], the first second of forced expiration [FEV_1_], and forced expiratory flow [FEF]) and preoperative PMI.

### Perioperative recipient and donor-graft factors

Preoperative recipient findings included age, body mass index (BMI), etiology of ESLD, comorbidities (diabetes mellitus [DM], systemic hypertension [HBP], diseases of the heart and kidney, lung disease determined by symptoms of dyspnea with atelectasis, consolidation, or pleural effusion using chest X-ray or CT images, smoking status, history of abdominal surgery, MELD score, complications of ESLD (mild encephalopathy [West-Haven grade I or II] [20], varix, and ascites [>1 L]), transthoracic echocardiography (ejection fraction and diastolic dysfunction), and laboratory findings (hematocrit, creatinine, total bilirubin, sodium, potassium, albumin, ammonia, glucose, international normalized ratio [INR], and platelet count). Intraoperative recipient findings included surgical duration, CRRT, administration of a strong vasopressor (i.e., norepinephrine infusion ≥0.1 μg.kg^− 1^.min^− 1^), significant PRS [28], hourly fluid infusion, hourly urine output, total amount of blood product transfused (PRBCs, fresh frozen plasma [FFP], platelet concentrate [PC], single donor platelets [SDPs], and cryoprecipitate), mean laboratory values (lactate, glucose, and brain natriuretic peptide [BNP]), and mean arterial blood gas analysis values (pH, hemoglobin, PaO_2_, SaO_2_, and PaCO_2_). Donor-graft findings included age, sex, BMI, graft-recipient weight ratio (GRWR), steatosis, and total graft ischemic time.

### Clinical postoperative outcomes

Clinical postoperative outcomes included duration of ventilator support, and the incidence of re-intubation, pneumonia and early allograft dysfunction (EAD) in the ICU. EAD was defined as the presence of one or more of the following: total bilirubin ≥10 mg.dL^− 1^ or INR ≥ 1.6 on postoperative day 7; and AST or ALT ≥2000 IU.mL^− 1^ during the first week [31]. The total lengths of the ICU and hospital stays were compared between the immediate and conventional extubation groups.

### Statistical analysis

The perioperative recipient and donor-graft factors were compared between the immediate extubation and conventional groups using the Mann–Whitney *U* test and the *χ*^2^ test. The normality of the distribution of continuous data was analyzed using the Shapiro–Wilk test. The OR extubation rates were compared among five expert anesthesiologists using the *χ*^2^ test. The correlations of spirometry parameters and preoperative PMI were evaluated using Spearman’s method. Perioperative factors affecting immediate extubation in the OR were analyzed using univariate and multivariate logistic regression. Significant factors (*p* < 0.1) in the univariate analysis were entered into the forward and backward multivariate analyses. The most relevant clinical factors were determined when multiple perioperative factors were correlated. The PMIs were compared between the two groups using the Mann–Whitney *U* test. In addition, the accuracy of the predictive model was analyzed using the area under the receiver operating characteristic curve (AUC). An optimal cut-off value of preoperative PMI according to OR extubation was determined using the AUC method. Values are expressed as means ± standard deviation (SD), medians and interquartile ranges (IQR), or as numbers and proportions. All tests were two-sided, and a *p*-value < 0.05 was considered significant. Statistical analyses were conducted using SPSS for Windows (ver. 24.0; SPSS Inc., Chicago, IL, USA) and MedCalc for Windows software (ver. 11.0; MedCalc Software, Ostend, Belgium). The TRIPOD reporting guidelines were followed during the development of the prediction model, and in the validation study [32].

## Results

### Baseline characteristics of the study population

The initial study population consisted of 295 male adult patients (age ≥ 19 years) who underwent LDLT at our hospital between January 2011 and December 2017. After removing 40 patients based on the exclusion criteria, 255 male patients who underwent elective LDLT remained. The average age of the patients was 52 ± 8 years and the BMI was 24.8 ± 3.6 kg.m^− 2^. The most common etiology was hepatitis B (66.3%), followed by alcohol (20.8%), hepatitis C (5.5%), toxin or drug (3.9%), hepatitis A (1.2%), autoimmune (0.4%), and cryptogenic hepatitis (2.0%). The median (IQR) MELD score was 13 (8–21) points and hepatic decompensation signs were as follows: ascites > 1 L (41.2%), encephalopathy (West-Haven grade I or II) (27.1%), varix (23.5%), and hepatorenal syndrome (12.2%). The average preoperative PMI was 359.4 ± 95.8 mm^2^.m^− 2^. In total, 101 of 255 patients (39.6%) were extubated in the OR immediately after surgery. There were no significant differences in OR extubation rate among the attending transplant anesthesiologists (Additional file 1).

Based on OR extubation, the optimal cut-off value of preoperative PMI was 352.2 mm^2^.m^− 2^ (AUC: 0.862; 95% confidence interval: 0.814–0.902; *p* < 0.001). Thus, 125 patients (49.0%) had non-sarcopenic features and 130 (51.0%) showed sarcopenic features (Additional file 2).

### Correlation between respiratory performance and preoperative PMI

Additional file 3 shows that preoperative PMI was weakly correlated with respiratory performance parameters, such as FVC (L), FVC (%), FEV_1_ (L), FEV_1_/FVC (%), FEF_25–75%_ (L.sec^− 1^), and FEF_75–85%_ (L.sec^− 1^). Only FEV_1_ (%) was moderately correlated with the PMI.

### Comparison of clinical characteristics between the immediate and conventional extubation groups

The preoperative recipient findings differed between the two groups, including with respect to BMI; incidence of lung disease; MELD score; the incidence rates of encephalopathy (West-Haven grade I or II) and ascites (>1 L); and the levels of hematocrit, sodium, albumin, platelets, total bilirubin and INR (Table 1).

**Table 1:** Comparison of preoperative recipient findings between the conventional and immediate extubation groups

Group	Conventional extubation	Immediate extubation	*p*
n	154	101	
*Preoperative recipient findings*			
Age (year)	53 (47–59)	54 (49–57)	0.983
Body mass index (kg.m^−2^)	23.9 (21.9–26.6)	24.9 (23.2–26.7)	0.016
*Etiology of end-stage liver disease*			
Alcohol	33 (21.4%)	20 (19.8%)	
Hepatitis A	2 (1.3%)	1 (1.0%)	
Hepatitis B	98 (63.6%)	71 (70.3%)	
Hepatitis C	11 (7.1%)	3 (3.0%)	
Autoimmune	0 (0.0%)	1 (1.0%)	
Toxin & drug	7 (4.5%)	3 (3.0%)	
Cryptogenic	3 (1.9%)	2 (2.0%)	
*Comorbidity*			
Diabetes mellitus	42 (27.3%)	19 (18.8%)	0.121
Systemic hypertension	32 (20.8%)	17 (16.8%)	0.434
Heart disease	7 (4.5%)	1 (1.0%)	0.152
Lung disease	25 (16.2%)	7 (6.9%)	0.028
Kidney disease	19 (12.3%)	6 (5.9%)	0.093
Current smoker	34 (22.1%)	20 (19.8%)	0.664
History of abdominal surgery	33 (21.4%)	13 (12.9%)	0.082
Model for end-stage liver disease (pts)	16 (10–26)	9 (7–14)	< 0.001
*Complications of end-stage liver disease*			
Mild encephalopathy	50 (32.5%)	19 (18.8%)	0.016
Varix	40 (26.0%)	20 (19.8%)	0.256
Ascites (> 1 L)	73 (47.4%)	32 (31.7%)	0.013
*Transthoracic echocardiography*			
Ejection fraction (%)	64.5 (62.0–67.9)	64.8 (62.0–67.0)	0.656
Diastolic dysfunction	52 (33.8%)	28 (27.7%)	0.379
*Laboratory findings*			
Hematocrit (%)	28.3 (24.4–33.9)	32.7 (26.6–38.2)	< 0.001
Creatinine (mg.dL^− 1^)	0.8 (0.7–1.2)	0.8 (0.7–0.9)	0.199
Total bilirubin (mg.dL^− 1^)	3.4 (1.0–14.5)	1.6 (0.7–4.2)	0.002
Sodium (mEq.L^− 1^)	138.0 (133.8–141.0)	141.0 (138.0–142.0)	< 0.001
Potassium (mEq.L^− 1^)	4.0 (3.7–4.3)	4.0 (3.8–4.3)	0.95
Albumin (g.dL^− 1^)	2.9 (2.7–3.5)	3.2 (2.7–3.7)	0.015
Ammonia (μg.dL^− 1^)	98.0 (65.0–159.0)	121.0 (73.3–178.0)	0.147
Glucose (mg.dL^− 1^)	113.5 (93.8–139.0)	101.0 (91.0–129.0)	0.053
International normalized ratio	1.6 (1.3–2.1)	1.4 (1.2–1.7)	0.004
Platelet count (× 10^9^.L^− 1^)	58.5 (42.0–87.3)	73.0 (48.0–124.0)	0.003

Values are expressed as medians (with interquartile range) or numbers (with % proportion)

The intraoperative recipient findings of the two groups also differed, in terms of surgical duration; frequency of administration of a strong vasopressor; incidence of significant PRS; hourly fluid infusion and urine output; requirement for blood products transfusion (i.e., PRBCs, FFP, PC, SDPs and cryoprecipitates); and the levels of BNP and hemoglobin (Table 2). Although SaO_2_ was lower in the immediate extubation group than in the conventional extubation group, the range of SaO_2_ was within normal limits (≥ 94%) in both groups [33]. Despite differences in the donor-graft findings, such as total graft ischemic time and GRWR, all transplant recipients received a graft of sufficient size (GRWR ≥0.8) [34].

**Table 2:** Comparison of intraoperative recipient and graft-donor findings between the conventional and immediate extubation groups

Group	Conventional extubation	Immediate extubation	*p*
n	154	101	
*Intraoperative recipient finding*			
Surgical duration (min)	515 (464–586)	490 (450–556)	0.031
Continuous renal replacement therapy	13 (8.4%)	3 (3.0%)	0.078
^a^Strong vasopressor administration	38 (24.7%)	11 (10.9%)	0.006
Significant postreperfusion syndrome	41 (26.6%)	5 (5.0%)	< 0.001
Hourly fluid infusion (mL.kg^−1^.h^− 1^)	10.9 (7.9–13.7)	9.3 (6.7–11.8)	0.001
Hourly urine output (mL.kg^− 1^.h^− 1^)	1.2 (0.6–2.0)	1.6 (0.9–2.3)	0.002
*Total amount of blood product transfusion during surgery (unit)*			
Packed red blood cell	9 (5–15)	4 (2–8)	< 0.001
Fresh frozen plasma	10 (5–13)	5 (3–7)	< 0.001
Platelet concentrate	0 (0–6)	0 (0–0)	< 0.001
Single donor platelet	0 (0–1)	0 (0–0)	0.003
Cryoprecipitate	0 (0–0)	0 (0–0)	0.001
*Average of laboratory factors during entire surgery*			
Lactate (mmol.L^− 1^)	4.4 (3.2–5.6)	4.2 (3.6–5.4)	0.85
Glucose (mg.dL^− 1^)	183 (156–206)	187 (163–206)	0.624
Brain natriuretic peptide (pg.mL^− 1^)	83 (40–172)	59 (31–103)	0.027
*Average of ABGA factors during entire surgery*			
pH	7.32 (7.28–7.37)	7.34 (7.31–7.38)	0.055
Hemoglobin (g.dL^− 1^)	9.5 (8.7–10.2)	10.5 (9.7–11.5)	< 0.001
PaO_2_ (mmHg)	189.4 (160.8–222.5)	187.0 (163.4–216.4)	0.971
SaO_2_ (%)	99.4 (99.0–99.6)	99.2 (98.8–99.5)	0.01
PaCO_2_ (mmHg)	36.0 (34.9–37.2)	36.0 (34.1–37.4)	0.76
*Donor-graft findings*			
Age (year)	33 (25–46)	32 (24–43)	0.923
Sex (Male)	89 (57.8%)	66 (65.3%)	0.227
Body mass index (kg.m^− 2^)	23.5 (22.1–25.1)	23.4 (21.0–25.0)	0.285
Graft-recipient weight ratio	1.2 (1.0–1.5)	1.1 (1.0–1.3)	0.019
Steatosis (%)	5.0 (0.0–5.0)	3.0 (0.0–5.0)	0.463
Steatosis type			0.264
None	51 (33.1%)	30 (29.7%)	
Microvesicular	13 (8.4%)	3 (3.0%)	
Macrovesicular	81 (52.6%)	62 (61.4%)	
Mixed	9 (5.8%)	6 (5.9%)	
Total graft ischemic time (min)	107 (74–147)	90 (66–113)	0.001

Values are expressed as medians (with interquartile range) or numbers (with % proportion)

*Abbreviation*: *ABGA* arterial blood gas analysis

^a^Strong vasopressor administration is defined as norepinephrine infusion ≥0.1 μg.kg^− 1^.min^− 1^

**Fig. 1:**
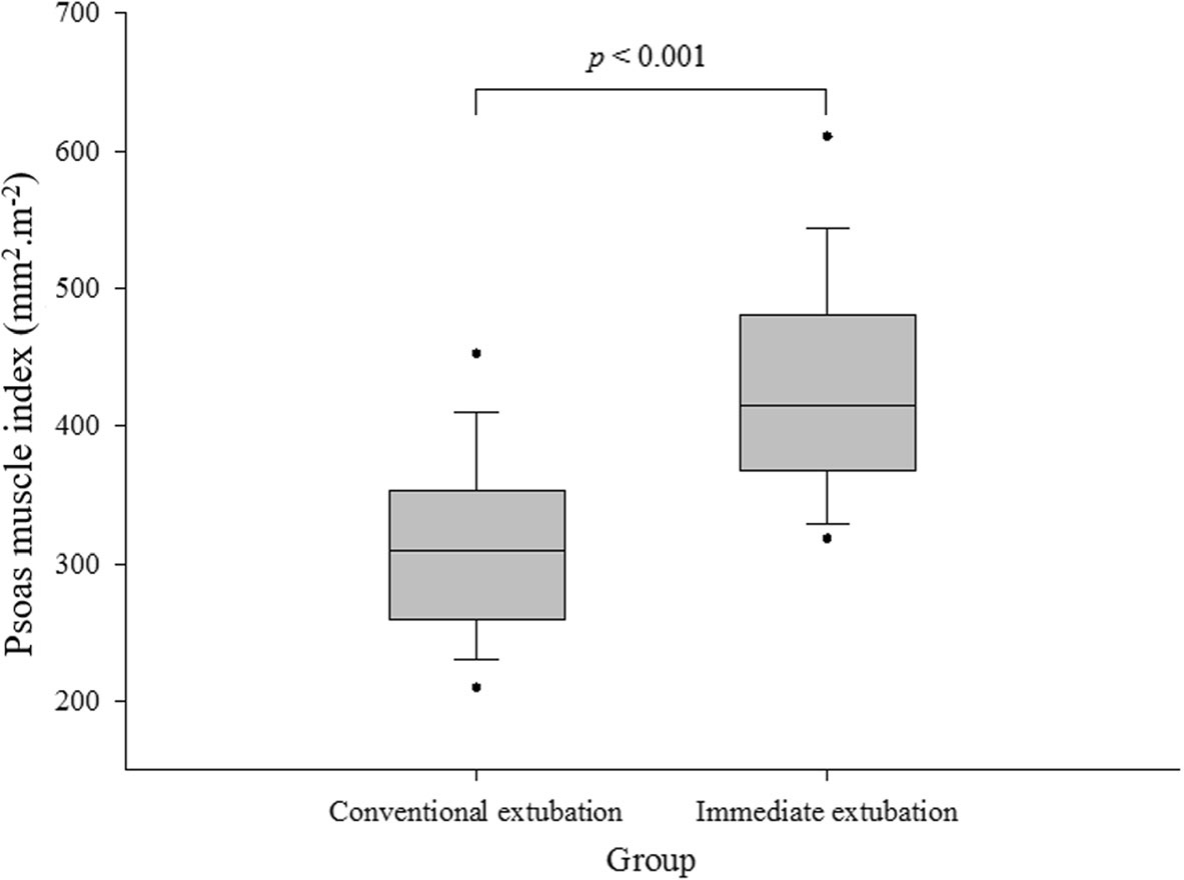
Comparison of preoperative psoas muscle index between the conventional extubation and immediate extubation groups in male patients who underwent elective living donor liver transplantation (LDLT). The box plots show the median (line in the middle of the box), interquartile range (box), 5th and 9^5^th percentiles (whiskers), and outliers (dots)

Preoperative PMI (median and IQR) was significantly higher in the immediate extubation group than in the conventional extubation group: 309.0 (259.6–352.7) mm^2^.m^− 2^ vs. 414.9 (367.7–480.0) mm^2^.m^− 2^ in the immediate extubation group (Fig. 1).

### Predictive factors for immediate extubation in the operating room

Table 3 suggests an association between perioperative recipient and donor-graft findings and immediate extubation in the OR among male patients who underwent elective LDLT. After an analysis of the potentially significant preoperative and intraoperative recipient and donor-graft findings in a multivariate logistic regression, the model revealed that preoperative PMI and intraoperative factors (i.e., use of CRRT, development of significant PRS, and FFP transfusion requirement) were independently associated with immediate extubation in the OR (AUC: 0.914; 95% confidence interval: 0.88–0.949; *p* < 0.001 in the predictive model).

**Table 3:** Association of perioperative recipient and donor-graft factors with early extubation in operating room in male patients undergoing elective living donor liver transplantation

	Univariate logistic regression				Multivariate logistic regression			
	*β*	OR	95% CI	*p*	*β*	OR	95% CI	*p*
*Preoperative recipient findings*								
Body mass index (kg.m^− 2^)	0.067	1.069	0.997–1.146	0.061				
Psoas muscle index (mm^2^.m^−2^)	0.02	1.02	1.015–1.025	< 0.001	0.025	1.025	1.017–1.033	< 0.001
Lung disease	−0.956	0.384	0.16–0.926	0.033				
History of abdominal surgery	−0.613	0.542	0.27–1.089	0.085				
Model for end-stage liver disease (pts)	−0.089	0.915	0.884–0.948	< 0.001				
*Complications of end-stage liver disease*								
Mild Encephalopathy	−0.73	0.482	0.264–0.88	0.018				
Ascites (> 1 L)	−0.664	0.515	0.304–0.87	0.013				
*Laboratory findings*								
Hematocrit (%)	0.07	1.072	1.032–1.114	< 0.001				
Total bilirubin (mg.dL^−1^)	−0.047	0.954	0.927–0.983	0.002				
Sodium (mEq.L^− 1^)	0.088	1.091	1.035–1.151	0.001				
Ammonia (μg.dL^−1^)	0.003	1.003	0.999–1.006	0.097				
International normalized ratio	−0.762	0.467	0.289–0.754	0.002				
Platelet count (× 10^9^.L^−1^)	0.006	1.006	1.002–1.011	0.007				
*Intraoperative recipient finding*								
Surgical duration (min)	−0.003	0.997	0.995–1.000	0.08				
Continuous renal replacement therapy	−1.103	0.332	0.092–1.196	0.092	−3.781	0.023	0.002–0.301	0.004
Strong vasopressor administration	−0.986	0.373	0.181–0.771	0.008				
Significant postreperfusion syndrome	−1.941	0.144	0.055–0.378	< 0.001	− 1.781	0.168	0.043–0.654	0.01
Hourly fluid infusion (mL.kg^− 1^.h^− 1^)	−0.105	0.901	0.847–0.958	0.001				
Hourly urine output (mL.kg^−1^.h^− 1^)	0.306	1.358	1.079–1.708	0.009				
*Total amount of blood product transfusion during surgery (unit)*								
Packed red blood cell	−0.123	0.885	0.841–0.931	< 0.001				
Fresh frozen plasma	−0.224	0.799	0.739–0.864	< 0.001	− 0.163	0.85	0.774–0.933	0.001
Platelet concentrate	−0.123	0.884	0.824–0.948	0.001				
Single donor platelet	−0.172	0.842	0.689–1.029	0.093				
Cryoprecipitate	−0.563	0.569	0.372–0.872	0.01				
*Average of ABGA factors during entire surgery*								
Hemoglobin (g.dL^−1^)	0.609	1.838	1.479–2.285	< 0.001				
SaO_2_ (%)	−0.313	0.731	0.508–1.051	0.091				
*Donor-graft findings*								
Graft-recipient weight ratio	−1.028	0.358	0.167–0.766	0.008				
Total graft ischemic time (min)	−0.011	0.989	0.983–0.995	< 0.001				

*Abbreviations*: *CI* confidence interval, *ABGA* arterial blood gas analysis

### Comparison of postoperative outcomes between the immediate and conventional extubation groups

The length of ICU stay and duration of ventilator support were shorter in the immediate extubation group than in the conventional extubation group, and the incidence of pneumonia and EAD were also lower in the immediate extubation group (Table 4). Three patients in the immediate extubation group underwent re-intubation in the ICU. The causes of re-intubation in the immediate extubation group were development of graft dysfunction (*n* = 2 patients) and respiratory distress due to pneumonia (*n* = 1 patient). A total of 10 patients in the conventional extubation group had re-intubation due to graft dysfunction (*n* = 3 patients), respiratory distress related to pneumonia (*n* = 5 patients), and miscellaneous reasons (*n* = 2 patients).

**Table 4:** Comparison of postoperative outcomes between the conventional and immediate extubation groups

Group	Conventional extubation	Immediate extubation	*p*
n	154	101	
Hospital stay (day)	23 (21–31)	23 (21–31)	0.53
Intensive care unit stay (day)	7 (6–8)	7 (5–7)	0.008
Ventilator support duration (min)	524 (225–764)	0 (0–0)	< 0.001
Re-intubation	10 (6.5%)	3 (3.0%)	0.211
Pneumonia	26 (16.9%)	4 (4.0%)	0.002
Early allograft dysfunction	26 (16.9%)	2 (2.0%)	< 0.001

Values are expressed as medians (with interquartile range) or numbers (with % proportion)

## Discussion

The main finding of this study was that preoperative PMI was an independent predictor of immediate extubation in the OR after elective LDLT, together with CRRT, significant PRS and a large FFP transfusion. The PMI value was higher in the immediate extubation group than in the conventional extubation group. Based on the standardized and universally accepted criteria for endotracheal extubation, the predictive accuracy of our model for OR extubation was high.

In our study, preoperative PMI positively correlated with respiratory performance quantified by spirometry parameters (i.e., FVC, FEV_1_, FEF_25–75%_, and FEF_75–85%_). This finding was supported by recent studies showing that preoperative muscular quantity and quality parameters (i.e., PMI and intramuscular adipose content [IMAC]) are associated with preoperative respiratory function parameters (i.e., vital capacity [VC] and FEV_1_) in patients who underwent hepatectomy for liver cancer [35]. In male patients who underwent LDLT, preoperative levels of PMI and IMAC, as well as grip strength (GS) were associated with preoperative VC and FEV_1_. In female patients, preoperative levels of IMAC and GS were associated with preoperative VC and FEV_1_ [29]. These studies suggested that patients with low muscular quantity and quality had poorer respiratory function than those with normal muscular quantity and quality [29, 35].

Our LDLT study suggested that increased preoperative core muscle mass (i.e., PMI) significantly increased the feasibility of OR extubation after elective surgery. Based on the standardized and universally accepted criteria for endotracheal extubation, sufficient core muscle mass before surgery seems to guarantee the recovery and maintenance of patient respiratory capacity immediately after surgery and improves the success rate of OR extubation without fatal complications. LDLT patients who are eligible for successful OR extubation may have sufficient physiological reserves to maintain homeostasis in the presence of external stress (i.e., surgery) whereas a low preoperative core muscle mass may be a marker of an increased risk of failed OR extubation [5, 36, 37]. In a surgical ICU study, muscle weakness in the extremities, measured as the ability to move against gravity and/or resistance, was associated with a higher re-intubation rate following extubation, with the additional consequences of prolonged mechanical support and weaning failure [38]. The MELD score is of limited value in reflecting the nutritional and functional condition of patients with ESLD, and preoperative core muscle mass measurements may help to identify patients suitable for immediate OR extubation, preventing unnecessary ventilator support after transplantation surgery [1, 5–7, 13, 39].

Many studies have focused on preoperative hepatic decompensation or intraoperative hemodynamic instability for predicting early extubation after LT [14, 15, 18], and our results also show that intraoperative factors related to hemodynamic disturbance (i.e., the use of CRRT, the occurrence of significant PRS, and a requirement for a large FFP transfusion) were negatively associated with immediate extubation in the OR. A prospective study by Biancofiore et al. [14] suggested that patients suitable for immediate postoperative extubation were predominantly male, and that a MELD score of 11 points was the optimal cut-off for immediate postoperative extubation. The immediate postoperative extubation group suffered less severe intraoperative hemorrhage and showed a lower requirement for blood product transfusion than the non-extubation group. Immediately after liver graft reperfusion, patients in the immediate extubation group experienced low blood pressure that required a vasopressor less frequently than those in the non-extubation group. Skurzak et al. [18] devised a prognostic score for early extubation in the OR after LT that consisted of two major factors (intraoperative PRBC transfusion ≥7 units and lactate level ≥ 3.4 mmol.L^− 1^ at the end of surgery) and three minor factors (home vs. hospitalized patients before LT; surgical duration ≥5 h; and dopamine ≥5 μg.kg^− 1^.min^− 1^ or norepinephrine ≥0.05 μg.kg^− 1^.min^− 1^ at the end of surgery). Another LT study suggested that the possibility of early extubation (within 3 h after surgery) was affected by intraoperative blood transfusion volume, vital organ function (i.e., kidney, heart, and lung), and hepatic encephalopathy, but early extubation did not correlate with patient age or severity of liver disease (i.e., United Network for Organ Sharing [UNOS] status and Child–Pugh classification) [15].

This study had some limitations. First, we were unable to investigate muscular strength because of the retrospective study design. GS was considered a useful proxy for muscular strength and was related to certain pulmonary parameters in previous studies [29, 35]. Second, we only analyzed factors associated with OR extubation in male LDLT patients because there are differences in muscle size and strength between the sexes [6, 40]; therefore, further studies that include female LDLT patients are needed. The effect of sex-specific muscular features on recovery of respiratory ability in patients with ESLD immediately after surgery would be an interesting topic for study [40]. Third, we were unable to investigate the relationship between age-related core muscle loss and the possibility of immediate extubation in the OR, because our cirrhotic patients were in their late 40s and 50s. Because aging influences muscle strength and mass, an age-specific study of cirrhotic patients is required. Fourth, although there were no significant differences in the OR extubation rates among attending transplant anesthesiologists, there may be differences in OR extubation methods, because there is no consensus on the specific OR extubation criteria that should be applied for LDLT patients. Further studies that use a standardized protocol for OR extubation are needed. Finally, there were some differences in preoperative and/or intraoperative conditions between the two groups, so the possibility of selection bias was not totally excluded. Therefore, further prospective matched studies are required to determine whether OR extubation has positive effects on postoperative outcomes, and whether the PMI can stand as an independent major parameter to determine immediate extubation in the OR after LT surgery.

## Conclusions

Immediate tracheal extubation in the OR is safe and beneficial as part of a rapid recovery pathway after elective LDLT. However, as respiratory failure can occur postoperatively, it is important to accurately identify LDLT patients who are eligible for immediate extubation in the OR. Our study could improve the accuracy of prediction of immediate post-transplant extubation in the OR by introducing preoperative PMI into predictive models of patients who underwent elective LDLT. Eventually, a predictive model of early extubation in the OR, including preoperative levels of PMI and intraoperative hemodynamic factors (i.e., the use of CRRT, the development of significant PRS and a requirement for a large FFP transfusion), may help transplant clinicians to determine which patients are suitable for successful immediate OR extubation, and prevent inadequate ventilator care and unnecessary ICU administration after elective LDLT.

## Additional files

Additional file 1: Comparison of OR extubation rates among five anesthesiologists. (DOCX 20 kb)

Additional file 2: Comparison of PMI between patients with and without sarcopenic features. (DOCX 17 kb)

Additional file 3: Correlation of preoperative psoas muscle index with spirometry parameters. (DOCX 18 kb)

## Data Availability

The datasets used and/or analyzed during this study are available from the corresponding author on reasonable request.

## Abbreviations

BMI: Body mass index
BNP: Brain natriuretic peptide
CT: Computed tomography
DM: Diabetes mellitus
ESLD: End-stage liver disease
FFP: Fresh frozen plasma
GWRW: Graft-recipient weight ratio
HBP: Systemic hypertension
ICU: Intensive care unit
INR: International normalized ratio
LDLT: Living donor liver transplantation
LT: Liver transplantation
MELD: Model for end-stage liver disease
OR: Operating room
PC: Platelet count
PMA: Psoas muscle area
PMI: Psoas muscle index
PRBCs: Packed red blood cells
PRS: Post-reperfusion syndrome
SDP: Single donor platelet
