# Supplementary material for: Analysis of pre- and intraoperative clinical for successful operating room extubation after living donor liver transplantation: a retrospective observational cohort study
Source: BMC Anesthesiol. 2019 Jun 28;19:112. doi: 10.1186/s12871-019-0781-z (PMC6598245; doi:10.1186/s12871-019-0781-z)
Supplement: Supplementary file 1 — Comparison of OR extubation rates among five anesthesiologists. (DOCX 20 kb) [file 12871_2019_781_MOESM1_ESM.docx]

**Additional file**

| **Additional file 1.** Comparison of OR extubation rates among five anesthesiologists | | | |
| --- | --- | --- | --- |
| ^*^Anesthesiologist | Conventional extubation | Immediate extubation | *p* = 0.983 |
| A | 29 (58.0%) | 21 (42.0%) |  |
| B | 31 (60.8%) | 20 (39.2%) |  |
| C | 35 (63.6%) | 20 (36.4%) |  |
| D | 27 (60.0%) | 18 (40.0%) |  |
| E | 32 (59.3%) | 22 (40.7%) |  |
| **Abbreviations:** OR, operating room; ICU, intensive care unit  ^*^Five anesthesiologists specializing in liver transplantation anesthesia  **NOTE**: Values are expressed as numbers and proportions (%). | | | |
