# Supplementary material for: Analysis of pre- and intraoperative clinical for successful operating room extubation after living donor liver transplantation: a retrospective observational cohort study
Source: BMC Anesthesiol. 2019 Jun 28;19:112. doi: 10.1186/s12871-019-0781-z (PMC6598245; doi:10.1186/s12871-019-0781-z)
Supplement: Supplementary file 2 — Comparison of PMI between patients with and without sarcopenic features. (DOCX 17 kb) [file 12871_2019_781_MOESM2_ESM.docx]

**Additional file**

| **Additional file 2.** Comparison of PMI between patients with and without sarcopenic features | | | |
| --- | --- | --- | --- |
| **Group** | **Non-sarcopenia**  **(≥ 352.2 mm^2^.m^-2)^** | **Sarcopenia**  **(< 352.2 mm^2^.m^-2^)** | ***p*** |
| **n** | **125** | **130** |  |
| **PMI (mm^2^.m^-2^)** | 415.6 (381.2 – 467.2) | 291.8 (251.1 – 326.1) | <0.001 |
| **NOTE:** Values are expressed as the median and interquartile range. | | | |
