# Supplementary material for: Analysis of pre- and intraoperative clinical for successful operating room extubation after living donor liver transplantation: a retrospective observational cohort study
Source: BMC Anesthesiol. 2019 Jun 28;19:112. doi: 10.1186/s12871-019-0781-z (PMC6598245; doi:10.1186/s12871-019-0781-z)
Supplement: Supplementary file 3 — Correlation of preoperative psoas muscle index with spirometry parameters. (DOCX 18 kb) [file 12871_2019_781_MOESM3_ESM.docx]

**Additional file**

| **Additional file 3.** Correlation of preoperative psoas muscle index with spirometry parameters | | |
| --- | --- | --- |
|  | Preoperative psoas muscle index | |
| *Pulmonary function test* | Correlation coefficient | *p* value |
| *Spirometry parameters* |  |  |
| FVC (L) | 0.185 | 0.008 |
| FVC (%) | 0.238 | 0.001 |
| FEV_1_ (L) | 0.255 | <0.001 |
| FEV_1_ (%) | 0.303 | <0.001 |
| FEV_1_/FVC (%) | 0.118 | 0.092 |
| FEF_25-75%_ (L.sec^-1^) | 0.188 | 0.009 |
| FEF_25-75%_ (%) | 0.183 | 0.011 |
| FEF_75-85%_ (L.sec^-1^) | 0.081 | 0.269 |
| FEF_75-85%_ (%) | 0.085 | 0.245 |
| **Abbreviations:** FVC, forced vital capacity; FEV_1_, the first second of forced expiration; FEF, forced expiratory flow | | |
